# Psycho-social components determining the strategies of coping with stress in undergraduate Polish nursing students

**DOI:** 10.1186/s12912-021-00630-2

**Published:** 2021-07-12

**Authors:** Iwona Bodys-Cupak, Anna Majda, Anna Kurowska, Ewa Ziarko, Joanna Zalewska-Puchała

**Affiliations:** Department of Nursing Fundamentals, Faculty of Health Sciences, Institute of Nursing and Midwifery, Jagiellonian University Medical College, 12 Michałowskiego Str, 31-126 Krakow, Poland

**Keywords:** Nursing, Student, Coping, Self-efficacy, Stress, Life satisfaction, Life optimism, Satisfaction, Support, Clinical learning environment

## Abstract

**Background:**

In order for the nursing students to fully benefit from the clinical experience, it is necessary for them to be able to handle education-related stress. It is important to establish the importance of personal resources and social determinants, that influence coping strategies in stressful situations.

**Methods:**

The cross-sectional study was conducted among 307 nursing students. The study research tools: Perceived Stress Scale (PSS-10), Life Orientation Test - Revised (LOT-R), Generalized Self-Efficacy Scale (GSES), Satisfaction With Life Scale (SWLS), Clinical Learning Environment Inventory (CLEI – 19), Brief COPE and the original questionnaire were used.

**Results:**

Active strategies of coping with stress were used significantly more often by the respondents with a greater sense of self-efficacy, a greater sense of life satisfaction and disposable optimism. Avoidance strategies of coping with stress were used significantly more often by the respondents with low self-efficacy, low level of disposable optimism, low sense of life satisfaction, and not a lot of teacher support. The higher was the intensity of stress experienced by students, the more often they coped by avoidance behaviour or showing helplessness.

**Conclusions:**

All the variables had a significant impact on coping with stress: the level of perceived stress, disposable optimism, sense of self-efficacy, sense of life satisfaction, as well as satisfaction with the stay in a hospital ward, support received from the teacher and the year of the study. The results of the research have allowed the identification of the needs in the field of strengthening the personal resources of nursing students. In the future, they may be useful for the development of educational programs.

## Background

In the last decades, awareness has grown that care over nursing students is crucial not only for their well-being and academic achievement, but is also essential in the process of preparing them to become professionals caring for others. Broadly understood emotional, informative, and instrumental support provided to future medical staff by teachers may have a positive impact on their psychosocial resources and thus effectively motivate them to self-develop and shape soft skills that play a large role when it comes to teamwork. Constructive interactions between university employees and students and a sense of belonging are conducive to launching various sources of support for people in difficult/problematic/stressful situations especially during education in a clinical environment [1].

It is characteristic for the education system to enforce the progress in learning in terms of knowledge and skills. It is inextricably linked with the stress experienced by the students. Every student experiences stress from the beginning of education [2]. Nursing students deal with various difficult situations, especially during practical classes in health care facilities [3]. Many studies have confirmed the occurrence of such stressful situations [4–9].

During undergraduate studies, nursing students in Poland spend over 50% of the hours of the 4800-h education program in health care units. They are exposed to various stressors directly related to the clinical learning environment. i.e. patient care, including, in particular, the performance of the caring activities and medical procedures, contact with the patient’s family, and cooperation with members of the interdisciplinary team.

Lazarus and Folkman (1984) stress theory was chosen as the guiding concept for the present study because it describes the nature of stress and the relationship between stress, coping and the environment [10]. Coping with stress is a positive factor that leads to growth and development, and stimulates new ventures. Coping with stress in the concept of R. Lazarus and co-authors is “the cognitive and behavioural efforts of the subject aimed at meeting specific external and/or internal requirements that are assessed by the subject as checking or exceeding personal resources” ([11], pp.572). This means that the specific remedial activity of a person is a consequence of the assessment of the situation and one’s own abilities. Therefore, the choice strategy of coping with stress is the result of the analysis of the situation and dispositional features referred to as styles [12]. Carver, Scheier, and Weintraub were the first to distinguish between the strategy and style of coping with stress – they treat strategy as situational and style as dispositional coping [13] This study focused on the assessment of situational coping with stress.

The methods of coping with stress applied by the students in stressful situations change in the education process [14]. Research has shown that students use various coping strategies from avoidance behaviours to strategies based on active action, e.g. problem solving [7, 15, 16]. Lack of skills related to coping with stress can cause depression, anxiety, behavioural problems, irritability, social withdrawal, and physical illness [5, 17–20]. According to Juczyński, the course of the process of coping with stress is determined by the personal resources possessed and the social support network [21].

Self-efficacy, level of disposable optimism, and life satisfaction are factors that correlate with the way of coping with stress. Self-efficacy has been recognized as a variable that moderates stress and methods of coping with it [22, 23], as well as preventing professional burnout [24]. Research by Delany et al. [25] suggests that enhancing positive coping strategies is an important aspect of building self-efficacy and cognitive control, as well as greater self-awareness of students as learners and future professionals. The students’ self-efficacy and choice of strategies of coping with stress are related to resilience and professional burnout.

The impact of optimism on coping with stress and quality of life has been described in earlier studies by Chang and Chan [26], as well as Cruz et al. [27]. In other words, that faith in achieving a goal promotes active forms of coping, while a lack of faith in success leads to abandonment of action.

An important element shaping the ways of coping with stress is the learning environment, especially the clinical environment. Students’ satisfaction with the classes taking place in the hospital ward depends on the stress they experience and the ability to cope with it [28, 29]. Life satisfaction is, in turn, a factor dependent on how nursing students cope with stress, as shown in research by Lardier et al. [29].

The support provided by the teacher during classes in the clinical environment determines the learning outcomes achieved by students and shapes attitudes towards the future profession [30]. Properly provided support significantly helps students reformulate the experience, facilitates the flexible use of internal and external coping resources, and allows them to reflect on themselves and take care of themselves, all of which help to increase their resilience [31].

Due to the fact that the effectiveness of each person is largely dependent on the intensiveness of stress, it can also be said that the ability to cope with stress is an important factor influencing the achievement of success in education. In Polish literature, there are no studies on the impact of the culture/environment of upbringing and teaching on the shaping of strategies for coping with stress among nursing students, therefore the present research was undertaken. The results of the research conducted among nursing students will allow for the verification of previous research results, finding differences, and the presentation of the issue in a different socio-cultural context.

## Methods

### Study design

The research was a cross-sectional study, carried out using a diagnostic survey, estimation, and scaling method. The aim of the study was to assess the impact of selected personal variables (sense of optimism, self-efficacy, life satisfaction) and satisfaction with clinical classes (teacher support, satisfaction with staying in a hospital ward) as well as the intensity of perceived stress and the year of the study, on strategies of coping with the stress of nursing students during clinical classes.

### Setting & participants

The study used a purposeful selection of participants to the study group. The research was conducted among full-time undergraduate nursing students at the Jagiellonian University Medical College (a leading academic centre in southern Poland). Sample size was calculated on G*Power 3.1.9.2, which revealed that a minimum of 207 participants was needed to perform a regression analysis with a median effect size of 0.15, significance level of 0.05, statistical power of 0.95.

### Measurements tools

The research used: Life Orientation Test - Revised (LOT-R), Generalized Self-Efficacy Scale (GSES), Satisfaction With Life Scale (SWLS), Clinical Learning Environment Inventory (CLEI-19), and Perceived Stress Scale (PSS-10), Brief COPE and questionnaire developed by members of the research team, which included among others questions about socio-demographic variables.

*Life Orientation Test - Revised* (LOT-R) by Scheier, Carver and Bridges [32]. The adaptation to Polish conditions was made by Poprawa and Juczyński. The LOT-R tool consists of the 10 items, 3 items measure optimism, 3 items measure pessimism, and 4 items serve as fillers. Respondents rate each item on a 4-point scale: 0 = strongly disagree, 1 = disagree, 2 = neutral, 3 = agree, and 4 = strongly agree. The respondent can get from 0 to 24 points. The higher the result, the higher the level of disposable optimism presented by the respondent. Raw results are analysed in relation to the sten scale, where a score of 1–4 is low (indicating a tendency towards pessimism), and the score of 7–10 is high (tendency towards a positive attitude). For the original version of the scale, the Cronbach’s alpha coefficient is 0.78, meanwhile, for the Polish version of the scale, it was similar and was equal to 0.79 [21].

*Generalized Self-Efficacy Scale* (GSES) measures the strength of an individual’s general faith in the effectiveness of coping with difficult situations and obstacles. The tool was made by Schwarzer and Jerusalem [33]. The tool was adapted to Polish conditions by Juczyński. The scale consists of 10 statements related to various personal characteristics, which the respondent assesses as true or false in relation to himself / herself using the following scoring: 1 - “no”. 2 - “rather not”. 3 - “rather yes”. 4 - “yes”. Raw results are analysed in relation to sten norms: 1–4 (low score), 5–6 (average score), 7–10 (high score). The theoretical range of the scale was 0 to 40 points. The higher scores corresponded to higher self-efficacy. The scale is characterized by a moderately high critical accuracy and reliability. Cronbach’s alpha coefficient for the scale is 0.85. meanwhile, for the Polish version, it was equal to 0.78 [21].

*Satisfaction With Life Scale* (SWLS) by Diener, Emmons, Larson and Griffin [34]. Polish adaptation of the tool has been made by Juczyński. By means of this tool, it is possible to assess the general index of the person’s satisfaction with life. Life satisfaction is expressed in the feeling of being satisfied with one’s own achievements and conditions. The scale consists of five statements rated on a seven-point scale. The respondent assesses to what extent each statement relates to his/her life and to what extent he/she agrees with each statement in accordance with scoring from 1 (“I completely disagree”) to 7 (“I completely agree”). The respondent can score from 5 to 35 points. The higher the score, the higher the sense of his/her life satisfaction. The scoring can be converted into sten scores, where scores from 1 to 4 are considered low, 5–6 average. and 7–10 high. Cronbach’s alpha coefficient for the scale is 0.85 meanwhile, for the Polish version of the scale it was equal to 0.81 [21].

*Clinical Learning Environment Inventory* (CLEI – 19).

The CLEI-19 questionnaire by Salamonson et al. [35] allows the assessment of the support from the teacher perceived by the student in terms of learning and his student satisfaction with the work in the hospital ward. The inventory contains 19 statements, out of which 12 statements are related to the teacher support and 7 of them to satisfaction with the work in the hospital ward. In each statement, the respondent decides how much the statement is consistent with his/her feelings by selecting answers from 1 (Strongly disagree) to 5 (Strongly agree) on a 5-point Likert scale. CLEI-19 total scores range from 19 to 95, with higher scores representing a more positive perception of the clinical learning environment [35]. Cronbach’s alpha coefficient of the original version of the scale is 0.93. In the Polish version of the scale which adaptation was carried out for the purposes of this study, Cronbach’s alpha coefficient is 0.92, for teacher support in the aspect of learning the Cronbach’s alpha coefficient is 0.95, whereas for satisfaction with hospital stay the Cronbach’s alpha coefficient is 0.90. The obtained scores indicate that the designed scale is a reliable tool (unpublished self-validation).

*The 10-item Perceived Stress Scale* (PSS-10) is used to assess the insensitiveness of stress related to life situation over the last month. The authors of the tool are Cohen, Kamarck and Mermelstein [36]. Adaptation of the scale to Polish conditions was carried out by Juczyński and Ogińska-Bulik. The scale contains 10 questions about subjective feelings related to personal problems and events. For each question the respondent chooses one of the answers on a 5-point scale (0 - “never”; 1 - “almost never”; 2 - “sometimes”; 3 - “quite often”; 4 - “very often”). The overall scale score is the sum of all points, the theoretical distribution of which ranged from 0 to 40. Scores ranging between 1 and 4 are considered low, scores between 7 and 10 high, and scores between 5 and 6 average. The higher the respondent’s score, the higher level of stress is experienced. The internal consistency of the original version of the scale for most subscales has an indicator close to 0.7. meanwhile, in the Polish version of the scale, it was 0.82 [37].

*Brief COPE* is used to assess the typical ways of reacting and feeling when experiencing stress and being in difficult situations. The tool created by Carver [13] and adapted by Juczyński and Ogińska-Bulik (called Inventory for Measuring Coping With Stress Mini-COPE) consisted of 28 theorems covering 14 strategies for coping with stress and difficult situations divided into 7 factors. i.e. Active Coping which includes the following strategies: Active coping, Planning, Positive reinterpretation; Seeking Support including Seeking emotional support and Seeking instrumental support; Helplessness including: Using psychoactive substances, Restraint coping, Self-blame; and Avoidance Behaviours including Finding other activities, Denial and Discharge, Turning to Religion, Acceptance, and Humour were treated as independent factors. For each statement the respondent selected one of four possible answers from “I almost never do this” (0 points) to “I almost always do this” (3 points). For each strategy, the score is calculated separately and the higher the score the more often the strategy is used. An in-depth analysis of the scores distinguishes three problem-focused strategies including Active Coping, Planning, Seeking Instrumental Support, and emotion-focused strategies covering Seeking Emotional Support, Turning towards Religion, and Denial. The reliability of the original version of the Inventory reaches indicators of nearly 0.70 for most scales [37].

The original questionnaire contained socio-demographic questions regarding among others: gender, age, and year of studies. It made it possible to collect information used for an in-depth characterization of the study group.

In this study, the dependent variable was the strategy of coping with stress, while the independent variables were: the level of dispositional optimism, the sense of generalised self-efficacy, the sense of life satisfaction, the intensity of the perceived stress, teacher’s support, satisfaction with the stay in the hospital ward, and the year of the study.

### Data collection

The study was conducted in the 2018/2019 academic year among people studying nursing at the first-degree level at the Faculty of Health Sciences Jagiellonian University Medical College.

At the design stage of the study, the criteria for inclusion in the study were established: studying nursing in full-time undergraduate studies, completion of clinical practice in hospital/clinic, and consent to participate in the study. The approval of the bioethics Committee of the Jagiellonian University was obtained prior to the research. The participants were informed about the confidentiality and anonymity of the study, that participation in the study was voluntary, and that it was possible to withdraw/refuse to cooperate at any stage of the study.

Questionnaires were distributed to the students during meetings that took place before the lectures. During the meetings, the participants were informed about the purpose of the study and the method of completing the questionnaire. Research team members distributed 450 questionnaires to all undergraduate students in the 2018/2019 academic year who met the inclusion criteria.

The participants of the study returned a total of 315 questionnaires (70% of all 450 questionnaires distributed). Eight questionnaires were rejected due to a significant lack of data, which made the analysis impossible. Ultimately, 307 questionnaires were used for the final analysis.

### Statistical methods

The analysis was performed with the use of IBM SPSS Statistics 20. The obtained raw results were entered into the database as coded data. During the analysis of the collected research material, statistical methods were used allowing for the development of results and conclusions. The analysis used the independence test chi^2^, the Kruskal-Wallis test, and the Spearman correlation coefficient. The choice of non-parametric tests was dictated by the lack of normal distribution of variables and the above was checked with the Kolmogorow-Smirnow and Shapiro – Wilk test. The comparison of the values of quantitative variables in the three groups was performed using the Kruskal-Wallis test. Correlation between quantitative variables was analysed using Spearman’s rank correlation coefficient.

The strength of dependence was interpreted according to the following formula: |r| ≥ 0.9 – very strong correlation; 0.7 ≤ |r| < 0.9 – strong correlation; 0.5 ≤ |r| < 0.7 – medium correlation; 0.3 ≤ |r| < 0.5 – small correlation; |r| < 0.3 – very small correlation (negligible). The study also used linear regression carried out with the stepwise method. Regression was calculated separately for each strategy of coping with stress. The obtained raw results were entered into the database as encoded data. A significance level of 0.05 was assumed [38].

### Ethical considerations

The research was approved by the Bioethics Committee – No. of approval: 072.6120.208.2018.

Students were informed of the confidentiality and anonymity of the study, that their participation was voluntary and that they may cease to cooperate at any time during the study.

The study was conducted in accordance with the principles of the Helsinki Declaration. All of the collected data are stored as protected files accessible according to the regulations of the General Data Protection Regulation [39, 40].

## Results

### Characteristics of the study group

The study group consisted of 307 people who met the inclusion criteria for the study, 96.1% of whom were women (*N* = 295) and 3.9% men (*N* = 12). The average age of the respondents was 20.82 ± 1.53. with their age ranging from 19 (*N* = 49) to 34 (*N* = 1). The average age of the women was 20.78 ± 1.33, while the average age of the men was 21.92 ± 4.06. 20- and 21-year-olds constituted the largest age groups (*N* = 90 i.e. 29.3% and *N* = 86. i.e. 28.0% respectively) 20.2% of the participants were 22 years old (*N* = 62), and few were slightly older than 20 years old (*N* = 20. i.e. 6.5%). Freshmen constituted 40.1% of all the respondents (*N* = 123), 37.5% of the participants were sophomores (*N* = 115) and 22.5% were juniors (*N* = 69) (Table 1).

**Table 1:** Sociodemographic data of the study group

Sociodemographic variables		N	%
Gender	woman	295	96.1
	man	12	3.9
Age (years)	20	90	29.3
	21	86	28
	22	62	20.2
	> 22	20	6.5
Year of the study	I	123	40.1
	II	115	37.5
	III	69	22.5

### The dispositional optimism of the students - LOT-R

A pessimistic attitude was presented by 30.0% of the students (*N* = 92). The average level of life orientation concerned 37.8% of the respondents (*N* = 116). In the case of 32.2% of people (*N* = 99) the life attitude was optimistic. The mean score of the LOT-R test was 14.43 ± 3.80 points and the scores were in the range of 2–24 points. Freshmen (38.21%) had a more optimistic attitude than other groups. The most pessimistic attitude among the respondents was presented by juniors (*p* = 0.017) (Table 2).

**Table 2:** The dispositional optimism of the students and the year of the study

			Year of the study			Total
			I	II	III	
**Life Orientation Test –Revised** (LOT-R)	Low scores	N	35	28	29	92
		%	28.5%	24.3%	42.0%	30.0%
	Average scores	N	41	47	28	116
		%	33.3%	40.9%	40.6%	37.8%
	High scores	N	47	40	12	99
		%	38.2%	34.8%	17.4%	32.2%
Total		N	123	115	69	307
		%	100.0%	100.0%	100.0%	100.0%
χ2 = 12.041; p = 0.017						

χ2 – chi^2^ independence test, p – significance level < 0.05.

### Generalized self-efficacy of nursing students’ - GSES

Based on the GSES scale, it was found that over half of the students (*N* = 179, i.e. 58.3%) had a high sense of self-efficacy. Average results were obtained by 34.2% of the respondents (*N* = 105), and low results by only 7.5% of the respondents (*N* = 23). High self-efficacy was demonstrated most often by sophomores (65.21%) and freshmen (62.6%), and only by 37.7% of the surveyed juniors. Low self-efficacy was demonstrated by 3.3% of freshmen, 6.1% of sophomores, and 17.4% of juniors. Average self-efficacy was demonstrated by 33.3% of freshmen, 28.7% of sophomores, and 44.9% of juniors. The differences were not statistically significant (*p* = 0.0002) (Table 3).

**Table 3:** Generalized self-efficacy of nursing students’ and year of the study

			Year of the study			Total
			I	II	III	
Generalized Self-Efficacy Scale (GSES)	Low scores	N	4	7	12	23
		%	3.3%	6.1%	17.4%	7.5%
	Average scores	N	41	33	31	105
		%	33.3%	28.7%	44.9%	34.2%
	High scores	N	78	75	26	179
		%	63.4%	65.2%	37.7%	58.3%
Total		N	123	115	69	307
		%	100.0%	100.0%	100.0%	100.0%
χ2 = 22.174; *p* = 0.0002						

*χ2* chi^2^ independence test, *p* significance level < 0.05

### The sense of satisfaction with the life of the surveyed nursing students - SWLS

A low level of satisfaction with life was declared by 27.0% of students (*N* = 83). Average scores were obtained by 42.0% of participants (*N* = 129) and high scores of 30.9% of respondents (*N* = 95). The average level of satisfaction with life was 20.82 ± 5.72 points and was in the range of 5–35 points. The average score obtained on the sten scale was 5.64 ± 2.00 points and was in the range of 1–10 points. A high level of satisfaction with life was demonstrated more often by sophomores (40.86%) than by other groups of respondents. The average level of satisfaction was shown more often by freshmen (45.52%) and juniors (42.02%). The differences were statistically significant (*p* = 0.0098) (Table 4).

**Table 4:** The sense of satisfaction with the life of the surveyed nursing students and the year of study

			Year of the study			Total
			I	II	III	
Satisfaction With Life Scale (SWLS)	Low scores	N	32	24	27	83
		%	26.0%	20.9%	39.1%	27.0%
	Average scores	N	56	44	29	129
		%	45.5%	38.3%	42.0%	42.0%
	High scores	N	35	47	13	95
		%	28.5%	40.9%	18.8%	30.9%
Total		N	123	115	69	307
		%	100.0%	100.0%	100.0%	100.0%
χ2 = 13.319; *p* = 0.0098						

*χ2* chi^2^ independence test, *p* significance level < 0.05

### Assessment of clinical learning environment - CLEI – 19

On the basis of the CLEI-19 scale, it was shown that the average summary score of teacher support in terms of learning was 31.73 ± 10.71 points and ranged from 12 to 53 points (scale range 12–60 points). Satisfaction with the stay in the hospital ward as a summary score amounted to an average of 26.22 ± 5.10 points and ranged from 11 points to 35 points. (scale range 7–35 points).

Taking into account the mean values of the two scales (1–5 point scale), it was possible to compare the scores. Thus, it was found that satisfaction with the stay in the hospital ward was higher (3.75 ± 0.73 points) than satisfaction with the teacher’s support in terms of learning (2.64 ± 0.89 points).

It has been shown that the higher the year of studies, the higher the teacher’s support factor in terms of learning. The lowest score was obtained by the freshmen (M = 1.68), while the sophomores (M = 3.28) and juniors (M = 3.30) scored almost twice as high (M = 3.30). The differences were statistically significant (*p* < 0.0001).

The level of satisfaction with staying in a hospital ward among freshmen was higher than for other surveyed groups (M = 4.36). Sophomores and juniors were less satisfied with their stay in the hospital ward (M = 3.33 in both groups). The scores were statistically significantly different (*p* < 0.0001) (Table 5).

**Table 5:** Assessment of teacher support and hospital stay satisfaction (CLEI-19) versus year of study

Year of the study		Teacher support	Satisfaction with clinical placement
I	M	1.68	4.36
	SD	0.42	0.49
II	M	3.28	3.33
	SD	0.41	0.51
III	M	3.30	3.33
	SD	0.42	0.60
Total	M	2.64	3.75
	SD	0.89	0.73
p		**< 0.0001**	**< 0.0001**

*M* mean, *SD* standard deviation, *p* significance level < 0.05

### Intensification of stress experienced by students - PSS – 10

The largest group among the respondents (*N* = 195, i.e. 63.5%) were students who experienced high levels of stress. The average level of stress was experienced by 28.7% of respondents (*N* = 88), and a low level of stress by 7.8% (*N* = 24). The mean score was 21.15 ± 5.30 points and ranged from 8 to 36 points. It was shown that the low level of stress was experienced more often by the freshmen (1st year =13.0%; 2nd year =6.1%; 3rd year =1.4%). The average level of stress was experienced more often by sophomores (32.2%) and juniors (33.3%) than freshmen (22.8%). A high level of stress was experienced by slightly more than 60% freshmen (1st year = 64.2%; 2nd year = 61.7%; 3rd year = 65.2%). The differences were statistically significant (*p* = 0.0281) (Table 6).

**Table 6:** Intensification of stress experienced by students and the year of study

			Year of the study			Total
			I	II	III	
Perceived Stress Scale (PSS-10)	Low scores	N	16	7	1	24
		%	13.0%	6.1%	1.4%	7.8%
	Average scores	N	28	37	23	88
		%	22.8%	32.2%	33.3%	28.7%
	High scores	N	79	71	45	195
		%	64.2%	61.7%	65.2%	63.5%
Total		N	123	115	69	307
		%	100.0%	100.0%	100.0%	100.0%
*p*			**0.0281**			

*p* significance level < 0.05

### Strategies of coping with stress used by nursing students - brief COPE

In difficult situations, students mainly chose the strategy of Active coping (2.10 ± 061) and Seeking Emotional Support (2.01 ± 0.76). To a slightly lesser extent, stressful situations were dealt with by Planning (1.96 ± 0.65) or Seeking Instrumental Support (1.95 ± 0.77). The least chosen strategy of coping with stress was the Use of Psychoactive Substances strategy (0.70 ± 0.92).

It was found that the year of studies had a significant impact on the ways of coping with difficult situations by the respondents. In comparison to freshmen and juniors - sophomores were more often, choosing strategies based on active coping (2.25 points), planning (2.09 points), positive reinterpretation (1.84 points), acceptance (1.95 points), and seeking emotional support (2.14 points), doing something else (2.00 points). Juniors, on the other hand, more often than other groups were choosing strategies based on denial (1.22 points), discharge (1.70 points), Use of psychoactive substances (1.03 points), discontinuation of activities (1.25 points) ( Table 7).

**Table 7:** Strategies of coping with stress used by nursing students and the year of study

Strategy	Total		I year		II year		III year		*p*
	M	SD	M	SD	M	SD	M	SD	
Active Coping	2.10	0.61	2.07	0.63	2.25	0.59	1.92	0.57	**0.0005**
Planning	1.96	0.65	1.85	0.68	2.09	0.63	1.92	0.63	**0.0111**
Positive Reinterpretation	1.71	0.69	1.60	0.70	1.84	0.66	1.67	0.68	**0.0259**
Acceptation	1.77	0.69	1.63	0.70	1.95	0.67	1.74	0.65	**0.0005**
Sense of Humour	1.22	0.72	1.07	0.75	1.25	0.69	1.44	0.63	**0.0006**
Turning Towards Religion	1.38	0.92	1.27	0.91	1.48	1.01	1.41	0.79	0.2077
Seeking Emotional Support	2.01	0.76	1.98	0.74	2.14	0.79	1.87	0.73	**0.0236**
Seeking Instrumental Support	1.95	0.77	1.93	0.84	2.04	0.70	1.84	0.75	0.1659
Finding Other Activities	1.81	0.68	1.74	0.68	2.00	0.69	1.64	0.57	**0.0001**
Denial	1.04	0.76	1.01	0.79	0.96	0.78	1.22	0.66	**0.0191**
Discharge	1.54	0.68	1.45	0.71	1.53	0.66	1.70	0.65	**0.0403**
Use of Psychoactive Substances	0.70	0.92	0.65	1.02	0.55	0.79	1.03	0.89	**0.0004**
Cessation of Activity	0.92	0.77	0.84	0.78	0.82	0.71	1.25	0.76	**0.0001**
Self-blame	1.39	0.73	1.46	0.76	1.30	0.69	1.43	0.71	0.2782

*M* mean, *SD* standard deviation, *p* significance level < 0.05

Moreover, it was found that students chose, above all, strategies based on seeking support (M = 1.98; SD = 0.7) and active coping (M = 1.92; SD = 0.52), and then Acceptance of situation (M = 1.77; SD = 0.69), avoidance behaviour (M = 1.46; SD = 0.47) and turning to religion (M = 1.38; SD = 0.92). In difficult situations they reacted to the smallest extend with a sense of humour (M = 1.22; SD = 0.72) or helplessness (M = 1.00; SD = 0.58).

### Determinants of the strategies of coping with stress in the surveyed nursing students

Statistical analysis showed that:

- ➢ The higher the sense of the effectiveness of the students who were subject of the study, the more often they coped with active forms of coping with stress (rho = 0.343; *p* = 0.000), seeking support (rho = 0.2; *p* = 0.000) and accepted the situation (rho = 0.209; *p* = 0.000). The lower the sense of effectiveness of the respondents was, the more often they showed a sense of helplessness (rho = − 0.22; *p* = 0.000).
- ➢ The higher was the intensity of stress experienced by students, the more often they coped by avoidance behaviour (rho = 0.21; *p* = 0.000) or showing helplessness (rho = 0.2; *p* = 0.000). On the other hand, the lower the intensity of stress experienced by students, the more often they were undertaking active forms of coping (rho = − 0.16; *p* = 0.005).
- ➢ The higher the sense of satisfaction with the life of the surveyed students, the more often they coped by undertaking active forms of coping (rho = 0.304; *p* = 0.000) and seeking support (rho = 0.304; *p* = 0.000); while the lower the sense of satisfaction with life, the more often they showed helplessness in a difficult situation (rho = − 0.151; *p* = 0.008).
- ➢ On the basis of the analysis, it can also be concluded that the more optimistic the respondents had an attitude to life, the more often they undertook active forms of coping (rho = 0.322; *p* = 0.000) or seek support (rho = 0.222; *p* = 0.000), while the more their attitude to life was pessimistic, the more often they showed helplessness (rho = − 0.273; *p* = 0.000).
- ➢ The higher the level of satisfaction of the surveyed students with their stay in the hospital ward, the more often they coped by seeking support (rho = 0.128; *p* = 0.025). On the other hand, the lower the sense of satisfaction with the stay in the hospital ward, the more often they coped by the sense of humour (rho = − 0.173; *p* = 0.002) or showing helplessness (rho = − 0.114; *p* = 0.045) (Table 8).

**Table 8:** Variables determining strategies undertaken by nursing students to cope with stress

Coping strategy	Self-efficacy		Perceived stress	Life satisfaction	Dispositional optimism	Satisfaction with clinical placement	Teacher support
Active Coping	rho	0.337	−0.140	0.188	0.285	0.033	0.051
	*p*	**0.0000**	**0.0142**	**0.0009**	**0.0000**	0.5702	0.3742
Planning	rho	0.230	−0.106	0.216	0.213	0.109	−0.061
	*p*	**0.0000**	0.0628	**0.0001**	**0.0002**	0.0559	0.2872
Positive Reevaluation	rho	0.252	−0.135	0.312	0.258	0.142	0.055
	*p*	**0.0000**	**0.0178**	**0.0000**	**0.0000**	**0.0127**	0.3342
Acceptation	rho	0.209	−0.092	0.220	0.203	0.168	−0.067
	*p*	**0.0002**	0.1059	**0.0001**	**0.0003**	**0.0032**	0.2389
Sense of Humour	rho	−0.017	− 0.032	0.042	−0.119	0.100	−0.173
	*p*	0.7673	0.5756	0.4680	**0.0379**	0.0792	**0.0023**
Turning Towards Religion	rho	0.053	−0.041	0.075	− 0.015	0.106	− 0.074
	*p*	0.3575	0.4735	0.1928	0.7991	0.0644	0.1942
Seeking Emotional Support	rho	0.201	−0.048	0.280	0.184	0.071	0.102
	*p*	**0.0004**	0.3993	**0.0000**	**0.0012**	0.2125	0.0730
Seeking Instrumental Support	rho	0.162	−0.101	0.282	0.228	0.070	0.129
	*p*	**0.0044**	0.0764	**0.0000**	**0.0001**	0.2182	**0.0240**
Finding Other Activities	rho	0.224	0.115	0.031	0.036	0.165	−0.022
	*p*	**0.0001**	**0.0444**	0.5842	0.5320	**0.0037**	0.6993
Denial	rho	−0.109	0.139	−0.047	− 0.242	0.000	− 0.061
	p	0.0570	**0.0151**	0.4115	**0.0000**	0.9981	0.2838
Discharge	rho	0.019	0.178	0.054	−0.108	0.086	− 0.045
	*p*	0.7468	**0.0018**	0.3498	0.0579	0.1327	0.4348
Use Psychoactive Substances	rho	−0.097	0.033	−0.021	− 0.160	0.008	− 0.149
	*p*	0.0914	0.5622	0.7167	**0.0048**	0.8897	**0.0089**
Cessation of Activity	rho	−0.263	0.136	−0.060	− 0.206	0.085	− 0.127
	*p*	**0.0000**	**0.0173**	0.2967	**0.0003**	0.1377	**0.0259**
Self- blaming	rho	−0.140	0.300	−0.230	− 0.246	− 0.032	− 0.023
	*p*	**0.0143**	**0.0000**	**0.0000**	**0.0000**	0.5820	0.6860

*rho* Spearman’s rank correlation coefficient, *p* significance level *p* < 0.05

### Variables determining the choice of coping strategies of nursing students – linear regression

Linear regression using the stepwise method was calculated on the sums of individual scales. This applies to all scales, i.e.: GSES, SWLS, LOT-R, CLEI, PSS-10. For the Sense of Humour strategy, none of the explanatory variables were significant, and therefore there were no variables in the model.

Active coping was chosen significantly more often by students with a higher sense of self-efficacy (beta = 0.274) and a higher level of dispositional optimism (beta = 0.194). Planning was more often chosen by students of older years (beta = 0.116) with a higher sense of self-efficacy (beta = 0.194) and a higher sense of satisfaction with life (beta = 0.166). The positive reevaluation was a strategy that was more often used by students with a higher sense of life satisfaction (beta = 0.210), a higher level of dispositional optimism (beta = 0.150), and a higher sense of self-efficacy (beta = 0.143) who received more support from the teacher (beta = 0.130). Acceptance was a strategy that was more often chosen by students with a higher sense of life satisfaction (beta = 0.175), a higher sense of self-efficacy (beta = 0.152), who received more support from the teacher (beta = 0.156). Older students significantly more often coped with their sense of humour (beta = 0.199). Seeking emotional support was a strategy chosen more often by students with a higher level of life satisfaction (beta = 0.212), and a higher sense of self-efficacy (beta = 0.124). Seeking instrumental support was significantly more often chosen by students with a higher sense of life satisfaction (beta = 0.288). Focusing on doing something else as a coping strategy was chosen more often by younger students (beta = − 0.268), who experienced the higher intensity of stress (beta = 0.163), who received more support (beta = 0.364), and had a higher sense of self-efficacy (beta = 0.217). Students with a lower sense of dispositional optimism coped significantly more often by denial. (beta = − 0.233). The discharge strategy was significantly more often chosen by students of older years (beta = 0.127) with a higher level of life satisfaction (beta = 0.172) experiencing more intense stress (beta = 0.254). Older students (beta = 0.323) receiving less teacher support (beta = − 0.248) were significantly more likely to cope with the use of psychoactive substances. Cessation of activities was a coping strategy chosen more often by students of older years (beta = 0.138) with a lower sense of self-efficacy (beta = − 0.224). Students with a lower sense of dispositional optimism (beta = − 0.174) experiencing higher levels of stress (beta = 0.210) were significantly more likely to blame themselves. Due to the multiplicity of the factors compared, it was decided to present statistically significant variables in Table 9.

**Table 9:** Variables determining choice of coping strategy of the respondents

Coping strategies/variables		Non-standardized coefficient		Standardized coefficient	t	*p*
		B	SE	Beta		
Active coping	General Self-Efficacy Scale	0.040	0.008	0.274	4.809	**< 0.0001**
	Revised Life Orientation Test	0.031	0.009	0.194	3.415	**0.0007**
Planning	General Self-Efficacy Scale	0.030	0.009	0.194	3.249	**0.0013**
	Satisfaction With Life Scale	0.019	0.007	0.166	2.824	**0.0051**
	Year of studies	0.099	0.047	0.116	2.086	**0.0379**
Positive reevaluation	Satisfaction With Life Scale	0.025	0.007	0.210	3.504	**0.0005**
	Revised Life Orientation Test	0.027	0.011	0.150	2.485	**0.0135**
	Teacher’s support	0.008	0.003	0.130	2.466	**0.0142**
	General Self-Efficacy Scale	0.023	0.010	0.143	2.436	**0.0154**
Acceptance	Satisfaction With Life Scale	0.021	0.007	0.175	2.977	**0.0031**
	Teacher’s support	0.010	0.004	0.156	2.831	**0.0049**
	General Self-Efficacy Scale	0.025	0.010	0.152	2.584	**0.0102**
Sense of Humour	Year of studies	0.184	0.052	0.199	3.538	**0.0005**
Seeking Emotional Support	Satisfaction With Life Scale	0.028	0.008	0.212	3.581	**0.0004**
	General Self-Efficacy Scale	0.022	0.011	0.124	2.100	**0.0366**
Seeking Instrumental Support	Satisfaction With Life Scale	0.039	0.007	0.288	5.252	**< 0.0001**
Finding Other Activities	General Self-Efficacy Scale	0.035	0.009	0.217	3.848	**0.0001**
	Teacher support in terms of learning	0.023	0.005	0.364	4.215	**< 0.0001**
	Year of studies	−0.236	0.077	−0.268	−3.062	**0.0024**
	Perceived Stress Scale	0.021	0.007	0.163	2.951	**0.0034**
Denial	Revised Life Orientation Test	−0.047	0.011	−0.233	−4.190	**< 0.0001**
Discharge	Perceived Stress Scale	0.033	0.008	0.254	4.189	**< 0.0001**
	Satisfaction With Life Scale	0.021	0.007	0.172	2.825	**0.0050**
	Year of studies	0.113	0.049	0.127	2.300	**0.0221**
Use of Psychoactive substances	Year of studies	0.386	0.107	0.323	3.611	**0.0004**
	Teacher’s support	−0.021	0.008	−0.248	−2.781	**0.0058**
Cessation of Activity	General Self-Efficacy Scale	−0.040	0.010	−0.224	−3.994	**0.0001**
	Year of studies	0.137	0.056	0.138	2.465	**0.0143**
Self-blaming	Perceived Stress Scale	0.029	0.008	0.210	3.522	**0.0005**
	Revised Life Orientation Test	−0.033	0.011	−0.174	−2.919	**0.0038**

*B* non-standardized regression coefficient, *SE* standard error, *Beta* standardized regression coefficient, *t* test t, *p* significance level < 0.05

## Discussion

This study have attempted to evaluate how Polish students are coping with stress in terms of clinical learning. The nature and quality of the student’s clinical experience, besides the transfer of knowledge is extremely important during the process of educating nursing students and shaping professional competences.

Stress is present in the lives of students throughout the education process, especially in clinical training [15, 16]. More than half of the students in the presented study felt stress of high intensity. Similar results were obtained by other researchers [8, 9, 16, 41]. Students undertake differentiated strategies of coping with stress in difficult situations. In this study, the respondents mainly chose the strategies of Active Coping and Seeking Emotional Support, and to a lesser extent Planning or Seeking Instrumental Support strategies. These results are consistent with the results obtained by other researchers [41–43]. Polish students revealed a greater intensity of the assessed remedial and adaptation strategies, i.e. Active Coping and Planning, than students from Spain or Slovakia [44]. Students from the presented study, however, less frequently used the avoidance strategy based on Psychoactive Substances, which has negative consequences on health. These results are consistent with the conclusions of other authors [14, 45]. In contrast, students from Spain obtained higher intensity of the strategies assessed, i.e. Use of Psychoactive Substances, Cessation of Activity and Self-blame, than Polish and Slovakian students [44].

Research conducted by Fornès-Vives et al. [14] showed that coping mechanisms and personality changes experienced by nursing students throughout their degree program seem to mirror the professional competencies needed by future licensed nurses. The results of the research carried out by Kaneko and Momino [5] allowed for the conclusion that in difficult situations, students were inactive in seeking help for solving problems. However, in the studies [42], nursing students displayed avoidance behaviour in stressful situations.

The authors’ own research showed that students experiencing a higher level of stress more often coped with it by Finding other activities, Discharging, Cessation activity or Self-blame. These results are consistent with the results obtained by other authors [8, 45, 46].

It was found that the year of studies had a significant impact on the nursing students’ stress coping strategies. Second-year students, to a greater extent than first-year and third-year students, chose strategies based on Active Coping, Planning, and Positive Reevaluation.

Nursing students in their senior level [47, 48] and those with a high level of self-efficacy [49] tended to use a problem-solving approach.

People with a higher sense of self-efficacy more often coped with stress through Active Counseling, Planning, Positive Reevaluation, Acceptance, Seeking Emotional Support, and Seeking Instrumental Support, similarly to other studies [22, 50]. Dealing with stress depends largely on personality variables influencing the choice of coping strategies [51].

A high and average level of optimism was displayed by the majority of the surveyed nursing students. People with a higher level of dispositional optimism significantly more often coped with stress through Active Coping, Positive Reevaluation, Planning, Acceptance, Seeking Emotional Support, and Seeking Instrumental Support. These results are consistent with other authors’ results [45]. A low level of optimism in the surveyed nursing students was significantly associated with more frequent coping through Denial, Ceasing Action, Self-blame, or Use of Psychoactive Substances. Research by Soares et al. [52] confirms the existence of a relationship between low optimism and the use of psychoactive substances. However, a more *reactive emotion focused coping* approach, defined as a strong emotional reaction, distortion, and impulsivity, is associated with reducing satisfaction with school and life. Cruz et al. [27] as well as Sohl and Moyer [48] indicated that optimism and proactive coping with stress at work positively influenced the quality of life.

The studies of Yildirim et al. [42] show that support is a determinant of coping with stress in nursing students. They emphasize the legitimacy of effective interventions counteracting educational stress, which would not only favour the development of skills, but also reduce its negative health effects. Other authors also pointed to the significant importance of support in strengthening positive self-image [52, 53], or mobilization to fight stress [54, 55]. In the conducted research, it was unequivocally shown that students who felt greater support from the teacher significantly more often “looked for instrumental support”. The results of the research conducted by Siu Yin Ching et al. [31] indicated the need to support, in particular, students undertaking coping strategies based on Self-blame.

Students who were more satisfied with their stay in the ward significantly more often coped with stress through Acceptance, Finding Other Activities, and Positive Reevaluation. The existence of the relationship between stress experienced by students during academic education and coping with it and satisfaction with life was demonstrated by Lardier et al. [29].

### Limitations of the study

The main limitations of the study are related to areas of data collection methods. Data collection was undertaken at one point in time rather than longitudinally. The characteristics of the participants – young people, mainly women – could represent a bias.

The selection of the group of respondents was deliberate, the research was carried out at one university, the conclusions cannot be applied to the general population. More research is needed.

### Strengths of the study

The results of the multivariate analysis of the influence of selected variables on coping with stress in nursing students allowed for the identification of the determinants of coping with stress during clinical education especially in times of stress.

Understanding the influence of psychosocial components on the coping strategies of Polish nursing students is important for several reasons. Firstly, the ability to cope with stress, appropriately shaped throughout education can influence the development of a positive professional identity and attitudes towards a future profession. Secondly, ensuring the nursing students have favourable conditions for education, using and strengthening their personal resources can have an effect on the learning results and indirectly on the quality of their care and safety of patients. Additionally, the ability to cope with stress at work will be more effective in preventing burnout, to which nurses are vulnerable due to the nature of their profession.

## Conclusions

All the variables had a significant impact on coping with stress: the level of perceived stress, disposable optimism, sense of self-efficacy, sense of life satisfaction, as well as satisfaction with the stay in a hospital ward, support received from the teacher and the year of the study.

Active strategies of coping with stress were used significantly more often by the respondents with a greater sense of self-efficacy, a greater sense of life satisfaction, and disposable optimism. Students experiencing more teacher support significantly were more often seeking instrumental support. Avoidance strategies of coping with stress were used by the respondents with low self-efficacy, low level of disposable optimism, low sense of life satisfaction, and not a lot of teacher support. Students who did not receive a lot of support from the teacher and those with a low level of dispositional optimism significantly more often used psychoactive substances. The greater intensity of stress perceived by nursing students’ determined more frequent avoidance behaviour.

The results of the research have allowed the identification of the needs in the field of strengthening the personal resources of nursing students. In the future, they may form the basis for the development of educational programs aimed at developing coping skills in various groups of nursing students. More intervention studies on stress management are also needed.

## Data Availability

The datasets used and analysed during the current study are available from the correspondent author based on reasonable request.
